# Acid Phosphates

**Published:** 1886-01-20

**Authors:** 


**Acid Phosphates.**—A few words of warning against the phosphate of lime, rendered soluable by the aid of hydrochloric, lactic, or an excess of phosphoric acid, may not be in opportune, seeing that its use has become very general of late years. Does this preparation, does Horsford's "Acid phosphate," merit the vogue which it enjoys ? We think not. Cherry-Lestage fed two groups of Cobayes for two months and a half, one set with pure bran, the other with mixtures of bran and different acid-phosphate solutions of lime—the hydro-phosphate, bi-phosphate, glycero-phosphate, or lacto-phosphate—and found that while the weight of those fed on pure bran increased 167 grammes in ten weeks, that of those fed on three of the mixtures decreased 60 grammes, and of those under the lacto-phosphate mixture, 155 grammes.—*Investigator.*

